# Surveillance of the spread of avian influenza virus type A in live bird markets in Tripoli, Libya, and determination of the associated risk factors

**DOI:** 10.14202/vetworld.2022.1684-1690

**Published:** 2022-07-16

**Authors:** Abdulwahab Kammon, Mosbah Doghman, Ibrahim Eldaghayes

**Affiliations:** 1 Department of Poultry and Fish Diseases, Faculty of Veterinary Medicine, University of Tripoli, Tripoli, Libya; 2 National Research Center for Tropical and Transboundary Diseases, Alzintan, Libya; 3 Department of Microbiology and Parasitology, Faculty of Veterinary Medicine, University of Tripoli, Tripoli, Libya

**Keywords:** avian influenza, live birds market, risk factors, Tripoli

## Abstract

**Background and Aim:**

Studies on avian influenza virus (AIV) in Libya are few and limited. This study aimed to determine the presence of AIV in live bird markets (LBMs) in Tripoli and determine the risk factors associated with AIV spread .

**Materials and Methods:**

In total, 269 cloacal swabs were randomly collected from different bird species in 9 LBMs located in Tripoli and its surrounding regions. The target species were ducks, geese, local chickens, Australian chickens, Brahma chickens, turkeys, pigeons, quails, peacock broiler chicks, and pet birds. Total RNA was extracted from the swab samples and used for real-time polymerase chain reaction to detect AIV type A.

**Results:**

Of the 269 samples, 28 (10.41% of total samples) were positive for AIV type A. The LBMs with positive samples were Souq Aljumaa, Souq Alkhamees, Souq Althulatha, and Souq Tajoura. The highest percentage (35.71%) of AIV was recorded in Souq Aljumaa. Positive results for AIV type A were obtained primarily in three species of birds: Ducks (14/65; highest percentage: 21.5%), local chickens (12/98; 12.24%), and geese (2/28; 7.14%). Furthermore, the following three risk factors associated with the spread of AIV type A were identified: Time spent by breeders/vendors at the market (odds ratio [OR] = 11.181; 95% confidence interval [CI] = 3.827–32.669), methods used for disposing dead birds (OR = 2.356; 95% CI = 1.005–5.521), and last visited LBM (OR = 0.740; 95% CI = 0.580–0.944). Restricting the movement of poultry vendors from one market to another may protect against AIV spread.

**Conclusion:**

The findings of this study indicate the high risk of AIV spread in LBMs and highlight the need for continuous surveillance of LBMs across the country.

## Introduction

Avian influenza (AI), one of the most contagious diseases in birds, is caused by type A influenza virus. Type A viruses are classified into two subtypes based on their surface glycoproteins, namely, hemagglutinin (H) and neuraminidase (N). To date, 18 H-containing type A (HA) and 11 N-containing type A (NA) subtypes have been identified; of them, 16 HA and 9 NA serotypes have been isolated and characterized in various species of birds [1].

Depending on their pathogenicity, AI viruses (AIVs) are classified into two types: Highly pathogenic AIVs (HPAIVs), which cause severe disease and are associated with a high mortality rate, and low-pathogenic AIVs (LPAIVs), which cause asymptomatic or mild disease [1].

In Libya, the first case of infection caused by the LPAIV subtype H9N2 in commercial poultry was reported in 2006; however, the virus could not be isolated then. In 2013, LPAIV H9N2 was isolated and characterized [2]. The first case of HPAIV (H5N1) infection in backyard chicken purchased from a live bird market (LBM) was reported in Tobruk, Libya, in March 2014; however, the spread was controlled successfully [2].

Many developing countries have LBMs; the best place to purchase various avian species for consumption as a protein source or rearing, a popular hobby globally [3]. In some countries, LBMs usually have a continuous structure where various birds are housed until they are sold; the markets open daily or on specific days of the week [4].

LBMs selling various avian species, those not implementing all-in–all-out management practices, and those lacking biosecurity are the potential sources of AIVs causing several avian diseases, including AI. LBMs are the source of viruses responsible for several AI outbreaks worldwide. AIV is an endemic virus found in many LBMs in the United States [5]. LBMs pose a threat to public health as they can facilitate the amplification and dissemination of AIVs [6]. LBMs represent a crucial part of the poultry value chain as they facilitate connections among various poultry stakeholders by gathering them in one place [7].

The continuous circulation of AIVs among birds in LBMs may play a role in spreading AIV and enhancing transmission to commercial and household poultry [3]. Therefore, any measures taken to control AI should consider LBMs one of the primary factors for disease transmission [8]. However, despite their well-established role in disease transmission and evident threat to human health as a continuous source of the severe acute respiratory syndrome and influenza viruses, LBMs around the world are not likely to be abolished [9].

In LBMs, naive birds often come in contact with infected birds. The newly infected birds may be sold or returned to the same farm, which facilitates the ongoing transmission of AIV within LBMs as well as from LBMs to farms across various geographical locations [10].

Libya is currently at risk of infections caused by HPAIV; hence, there is an urgent need for the surveillance of AIV spread. To the best of our knowledge, no recorded information is available on the role of LBMs in the spread of AIVs in Libya. Therefore, the present study aimed to detect AIV in LBMs located in Tripoli and its surrounding regions; we aimed to identify the role of LBMs in AIV spread and determine the risk factors associated with AIV infection and transmission.

## Materials and Methods

### Ethical approval

No ethical approval was required as samples were collected for diagnostic purposes. However, samples were collected as per animal welfare using international standard sample collection methods.

### Study period and location

The study was conducted from February to March 2018. Chicken farms located in Gaser Bin Gasher area and nine LBMs in Tripoli were included for sampling.

### Sampling

In total, 269 cloacal swab samples were collected from various species of birds from 9 LBMs located in Tripoli and its surrounding regions. The LBMs assessed were Souq Tajoura, Souq Aljumaa, Souq Althulatha, Souq Alahad, Souq Alkhamees, Souq Alsaeh, Souq Janzour, Souq Suliman Khater, and Souq Alhot (each market was visited once). Cloacal swabs were collected and placed in a transport medium (phosphate-buffered saline containing 50% glycerol, 2000 U/mL penicillin, 250 mg/mL gentamicin, and 500 U/mL nystatin) in accordance with the Office International des Epizooties guidelines [11].

The target species were as follows: Duck, geese, local chickens, Australian chickens, Brahma chickens, turkeys, pigeons, quails, peacock broiler chicks, and pet birds (Table-1). If an owner did not permit the collection of cloacal swabs, fecal material was collected from the cages. The samples were analyzed using real-time reverse transcription-polymerase chain reaction (rRT-PCR) to detect AIV.

**Table 1:** The positive and negative results of the samples collected from LBMs located in Tripoli.

LBM	Poultry species	Number of samples	Laboratory result	

			Positive (%)	Negative (%)
Souq Alkhamees (n=41)	Ducks	16	6 (37.5)	10
	Goose	5	0	5
	Local chicken	10	3 (30)	7
	Australian chickens	6	0	6
	Turkey	3	0	3
	Peacock	1	0	1
Souq Aljumaa (n=30)	Ducks	18	8 (44.4)	10
	Goose	5	2 (40)	3
	Local chicken	3	0	3
	Turkey	4	0	4
Souq Althulatha (n=30)	Ducks	12	0	12
	Goose	3	0	3
	Local chicken	15	5 (33.3)	10
Souq Alsaeh (n=33)	Ducks	9	0	9
	Goose	9	0	9
	Local chicken	12	0	12
	Turkey	3	0	3
Souq Janzour (n=29)	Ducks	6	0	6
	Local chicken	13	0	13
	Broiler chicks	6	0	6
	Brahma	4	0	4
Souq Tajoura (n=27)	Local chicken	23	4 (17.4)	19
	Australian chickens	2	0	2
	Brahma	2	0	2
Souq Alahad (n=33)	Ducks	2	0	2
	Goose	4	0	4
	Local chicken	12	0	12
	Turkey	6	0	6
	Peacock	3	0	3
	Quail	6	0	6
Souq Alhot (n=29)	Ducks	2	0	2
	Goose	2	0	2
	Local chicken	6	0	6
	Pigeon	14	0	14
	Canary	3	0	3
	Budgerigar	2	0	2
Suliman Khater (n=17)	Canary	11	0	11
	Budgerigar	6	0	6
Total		269	28 (10.4)	241 (89.6)

LBMs=Live bird markets

### RNA extraction and rRT-PCR

Viral RNA was extracted using the NucleoSpin^®^ RNA Set for NucleoZOL (Macherey-Nagel, Duren, Germany). To detect AIV type A, a commercial rRT-PCR kit (Microboss Hightech GmbH, Germany) was used according to the manufacturer’s instructions.

### Questionnaire

A structured questionnaire comprising seven questions was developed to determine the risk factors associated with AIV infection in LBMs. A total of 45 participants (breeders/traders/owners) responded to the questions by visually inspecting the birds. The questions sought information on bird species, bird source, last visited LBM, time spent by breeders/vendors at the market, methods used for disposing of dead birds, LBM location, and the type of disinfectant used [12].

### Statistical analysis

Statistical analysis was performed using the Statistical Package for the Social Sciences (SPSS) program version 22 (IBM^®^ SPSS^®^, NY, USA). Laboratory and questionnaire data were stored in a Microsoft Excel spreadsheet. Univariate analysis was performed to determine the correlations between the factors and prevalence of AIV type A. In addition, logistic regression analysis was performed to identify risk factors associated with AIV infection in LBMs.

## Results

### Surveillance of AIV spread in LBMs using rRT-PCR

Of the 269 samples collected from the 9 LBMs, 28 tested positive for AIV type A, representing 10.41% of the total number of samples tested (Table-1). However, the AIV subtypes could not be identified. Positive results were obtained from 4 out of 9 (44.44%) LBMs surveyed. The LBMs from which positive samples were obtained were Souq Aljumaa, Souq Alkhamees, Souq Althulatha, and Souq Tajoura, accounting for 35.71%, 32.14%, 17.85%, and 14.5% of the total number of positive results obtained from all LBM samples, respectively. The highest percentage AIV infection was noted in Souq Aljumaa (Table-2).

**Table 2:** The percentage of positive AIV found in each market.

No.	LBM	Positive	Percentage (positive/total positive)
1	Souq Alkhamees	9	32.14
2	Souq Aljumaa	10	35.71
3	Souq Althulatha	5	17.85
4	Souq Tajoura	4	14.3
5	Souq Alsaeh	0	0
6	Souq Janzour	0	0
7	Souq Alahad	0	0
8	Souq Alhot	0	0
9	Suliman Khater	0	0
Total		28	100

AIV=Avian influenza virus

In this study, positive results for AIV were primarily obtained from three bird species. The highest percentage of AIV-positivity was detected in ducks (14/65; 21.5%), followed by local chickens (12/98; 12.24%) and geese (2/28; 7.14%; Table-3).

**Table 3:** The positive result of avian influenza in different species in live bird markets in Tripoli.

Species	Total number of samples	Laboratory result		Percentage of infected birds

		Positive	Negative	
Duck	65	14	51	21.5
Local chicken	98	12	86	12.24
Goose	28	2	26	7.14
Total	191	28	163	14.65

### Risk factors associated with AIV type A infection in LBMs

The following points were identified using the data obtained from the questionnaire completed by poultry owners, breeders, and traders:

1. All bird owners sell various species of birds in the same place without segregation or following hygiene protocols.
2. The sources of birds being sold in LBMs vary; 54% of the birds were found to be from the market itself, which suggests that breeders simultaneously buy and sell from one another in the same market without implementing any biosecurity measures.
3. Most breeders move across markets in the same selling period; in most cases, they sell birds from one market to another in < 24 h.
4. Most breeders return unsold birds directly to the respective source without implementing any biosecurity measures.
5. Breeders were questioned regarding the methods used for disposing of dead birds: 42% answered that they buried the dead birds on the farm; 21% burned them and buried the dead birds on the farm; 35% threw the dead birds away; and 2% burned the dead birds and threw them away.

The analysis of the questionnaire data revealed that 58% of the traders and owners mentioned that their birds did not have any history of illness during the past 3 months, whereas approximately 42% of the traders mentioned that their birds had exhibited moderate symptoms such as diarrhea and respiratory symptoms.

Univariate analysis of seven factors revealed that the factors associated with the spread of AI type A virus were as follows: Bird species (F = 4.51; p = 0.035), methods used for disposing dead birds (F = 14.44; p = 0.001), time spent by breeders/vendors at the market (41.15; p = 0.001), and LBM location (F= 8.30; p = 0.001). The following factors correlated negatively with AIV infection: Bird source, disinfectant use, and last market visited. However, multivariate logistic regression analysis showed that the spread of AIV type A was associated with three risk factors: Time spent by breeders/vendors at the market (odds ratio [OR] = 11.181; 95% confidence interval [CI] = 3.827–32.669), methods used for disposing dead birds (OR = 2.356; 95% CI = 1.005–5.521), and last visited LBM (OR = 0.740; 95% CI = 0.580–0.944; Table-4). The last visited LBM was identified as a potential protective factor against AIV spread; vendors carrying AIV-positive birds may move across LBMs as the samples that showed positive results on rRT-PCR were collected on different days (Table-5).

**Table 4:** Logistic regression analysis of risk factors for AIV contamination of LBMs.

Potential risk factors	OR	95% CI for OR	p-value
Time spent at the market	11.181	3.827–32.669	0.001
Disposal of dead birds	2.356	1.005–5.521	0.001
Last visited LBM	0.740	0.580–0.944	0.015

AIV=Avian influenza virus, LBMs=Live bird markets, OR=Odds ratio, CI=Confidence interval

**Table 5:** Movement of poultry vendors between LBMs.

LBM positive for AIV	LBM last visited by the vendor	Bird species	Time spent (h)
Souq Aljumaa	Souq Tajoura	Ducks and geese	4–5
Souq Athulatha	Souq Aljumaa	Local chickens	5–6
Souq Alkhamees	Souq Athulatha	Ducks and chickens	4–5
Souq Tajoura	Souq Athulatha	Local chickens	4–5

AIV=Avian influenza virus, LBMs=Live bird markets

## Discussion

In the present study, AIV-positive samples were detected in 4 out of 9 (44.44%) LBMs. LBMs facilitate the spread of AIV among poultry species and humans, particularly those who handle and sell birds in traditional bird markets [5, 6, 9]. Abdelwhab *et al*. [13] detected the presence of AIV in 5.6% of the LBMs they surveyed in the Canal region in Egypt. Helal *et al*. [8] reported a low AIV prevalence (4.3%) in LBMs in Egypt. Nitipan *et al*. [14] could not find any AIV-positive sample in Bangkok LBMs during their study period because AIV was not circulating at that time.

The percentages of AIV-positive samples obtained from the LBMs surveyed in the present study were 35.71%, 32.14%, 17.85%, and 13.4% for Souq Aljumaa, Souq Alkhamees, Souq Althulatha, and Souq Tajoura, respectively. These results are consistent with our other findings that the time spent by breeders/vendors at LBMs caused an 11-fold increase in the risk of AIV infection and transmission.

Regarding the poultry species, the highest numbers of positive cases were detected in ducks and local chickens, representing 21.5% and 12.24% of the total number of positive cases, respectively; the lowest number of positive cases was detected in geese (7.14%). In Vietnam, a recent study showed that the prevalence of AIV was 27.5% and 24.8% in chickens and ducks, respectively [15]. Another study in Cambodia reported that 20.0% and 32.6% of the samples obtained from chickens and ducks, respectively, in local markets were positive for AIV [16]. Regarding AIV H5N1, Songserm *et al*. [17] indicated that free-ranging ducks are a potential reservoir of the virus. Thus, ducks are considered to play a key role in transmitting the virus, with a potential consequence of disease in domestic poultry [18, 19]. Ducks are known as the waterfowl reservoir of AIV and may play an important role in spreading AIV infection worldwide [15, 18, 20]. Other risk factors observed in our study were keeping ducks and geese in the markets for sales. Our findings are comparable with those of other studies reporting that ducks and geese act as the natural reservoirs of AIV; in particular, the highest rate of virus isolation was noted in duck samples [21, 22]. Ducks and geese have been known as AIV carriers since a long time as they can be infected with the virus for a prolonged period without showing any clinical symptoms and may act as the source of infection by facilitating AIV spread to other avian species in LBMs [23, 24].

Furthermore, waterfowl may have a subclinical infection caused by AIV and help maintain the virus in these markets for longer periods. In our study, despite the correlation noted between bird species and AIV-positive results, it was not identified as a significant risk factor. Notably, ducks can excrete AIV for at least 2 weeks through the cloacal and respiratory routes [25]. Thus, keeping various species of birds together in LBMs provides appropriate conditions for the transmission of AIV among the poultry species. This result is supported by the fact that AIV detection rate is twice as high in domestic poultry compared with that in the same species of pet birds [26]. In addition, selling different species of birds together in markets and nonsegregated keeping of various bird species in the same cage enables cross-transmission of the virus among birds [22, 27]. Younjung *et al*. [28] stated that the silent transmission of AIVs within LBMs resulted from the overcrowding and continuous supply of various susceptible avian species.

Regarding the effect of season on AIV spread, the highest prevalence was noted in winter. Abdelwhab *et al*. [13] reported a high AIV-positivity in samples obtained from LBMs during the cold month of February. Our results might be attributed to the fact that AIV survival and viability are associated with the lower temperatures of the environment. Viral transmission is suppressed in summer because of hot weather and dryness. Thus, the disease is associated with cold weather because it provides favorable conditions for the amplification and spread of the virus [29, 30]. Sakoda *et al*. [31] and Choi *et al*. [32], respectively, reported a higher prevalence of H5N1 infection in Japan and Korea in wild and domestic birds in the winter season. The same finding was reported in Egypt; the risk of AI was higher in winter than in other seasons [33]. In contrast, increased temperature is a predictor of reduced AIV transmission and survival [23, 34, 35].

Concerning the geographical distribution of AIV in the Tripoli region, Kammon *et al*. [2] detected H5N1-positivity in samples obtained from Toubrok and surrounding regions. This may indicate the spread of AIVs across regions, although the present study did not identify the subtypes of AIV.

Biosecurity is a crucial measure for disease control. The non-implementation of biosecurity measures on several farms is a key factor for AI persistence. Knowledge about biosecurity is essential for reducing the likelihood of the transmission of transmissible diseases [36, 37]. The circulation of the AIVs in LBMs may be because of the non-implementation of biosecurity measures or absence of any veterinary supervision in these markets as well as the unhygienic transportation of birds across localities [8, 37]. In our study, the time spent by breeders/vendors in LBMs caused an 11-fold increase in the risk of AIV infection and transmission. In China, Wang *et al*. [4] identified several risk factors associated with the AI outbreak, including the time spent in the market per day, different live avian species in the market, location of LBM, and a number of birds in the market. A longer daily market period increases the chance of virus transmission [3, 4, 38–40]. One of the risk factors identified in our study was the methods used for disposing of dead birds, showing a 2.4-fold increase in risk when dead birds are disposed of in landfills.

## Conclusion

The study may serve as a basis for future studies classifying AIV strains and investigating the virulence characteristics in Libya to provide insights into the epidemiology of infectious diseases and molecular characterization of the strains. Furthermore, our findings highlighted the role of LBMs in the spread of AIV and, therefore, the need for future research. The detection of HPAIV and LPAIV using DNA sequencing is crucial; unfortunately, this was not performed in this study.

## Authors’ Contributions

AK and IE: Designed the study. MD: Visited live bird markets and collected swab samples. AK and IE: Data analysis, drafted, and revised the manuscript. All authors have read and approved the final manuscript.
